# The ubiquitin protease Ubp10 suppresses the formation of translocations at interstitial telomere-like sites

**DOI:** 10.1093/nar/gkaf1373

**Published:** 2025-12-17

**Authors:** David I Gonzalez, Allison R Westerbeek, Esther A Epum, Katherine L Friedman

**Affiliations:** Department of Biological Sciences, Vanderbilt University, Nashville, TN 37235, United States; Department of Biological Sciences, Vanderbilt University, Nashville, TN 37235, United States; Department of Biological Sciences, Vanderbilt University, Nashville, TN 37235, United States; Department of Biological Sciences, Vanderbilt University, Nashville, TN 37235, United States

## Abstract

Double-strand breaks (DSBs) pose a significant threat to chromosome stability and, if left unrepaired, can result in chromosome rearrangements. Canonical DNA repair pathways mitigate these risks. However, if these repair mechanisms fail to repair the DSB, alternative repair pathways, such as break-induced replication, single-strand annealing, and *de novo* telomere addition (*dn*TA), can be utilized. Yeast subtelomeric regions are hotspots of recombination, while interstitial telomere-like sites can promote *dn*TA. In yeast, *dn*TA sites, termed SiRTAs (sites of repair-associated telomere addition), require Cdc13 association. We identified the ubiquitin protease Ubp10 as a positive regulator of *dn*TA at SiRTAs. Loss of *UBP10* reduces *dn*TA frequency but increases the frequency of other chromosomal rearrangements at SiRTAs. SiRTAs utilize the repetitive subtelomeric regions of donor chromosomes to facilitate rearrangements, with a fraction occurring independently of *RAD51* and requiring Sir4 and Sir2 components of the SIR complex. A DNA sequence capable of binding Cdc13 is necessary and sufficient to stimulate translocations in the absence of *UBP10*. This study highlights the diversity of DNA repair mechanisms at SiRTAs, advancing our understanding of telomere maintenance and chromosomal rearrangement formation.

## Graphical Abstract

**Graphical Abstract:**
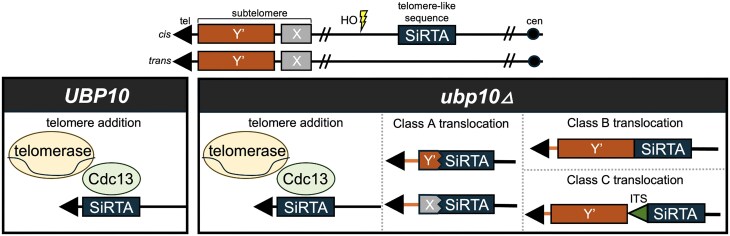

## Introduction

The maintenance of genomic integrity is crucial for human health, as alterations in the genome can lead to cancer or inherited genetic disorders. Eukaryotic chromosomes terminate in specialized nucleoprotein structures called telomeres, which play an essential role in maintaining genomic stability and integrity [1]. Telomeres distinguish natural chromosome ends from processed DNA double-strand breaks (DSBs), ensuring proper cellular responses to DNA damage [2]. Telomeres also serve as primers for elongation by the ribonucleoprotein enzyme complex telomerase, counteracting the sequence loss inherent to each replication cycle [3]. Telomerase, a reverse transcriptase, utilizes an intrinsic RNA component as a template to extend the TG-rich sequence (TG_1–3_ in yeast) of the 3′ single-stranded DNA (ssDNA) overhang at the chromosome terminus [1, 2, 4]. Subsequently, the CA-rich strand is synthesized by the lagging strand polymerase machinery [5]. In budding yeast, the ssDNA binding protein Cdc13 recruits and coordinates the actions of both telomerase and the lagging strand machinery [2, 6, 7]. Sequence-specific recognition of the telomeric 3′ overhang by Cdc13 is required for telomere integrity and replication.

DSBs represent one of the most severe threats to genomic stability. Unrepaired DSBs can lead to mutations, chromosomal rearrangements, or cell death. Cells employ canonical DNA repair pathways to address these breaks, including homologous recombination (HR) and non-homologous end-joining (NHEJ) [8]. NHEJ directly ligates the broken DNA with minimal processing, while HR utilizes a homologous template for accurate repair. For HR to proceed, the 5′ ends at a DSB must be resected to generate 3′ ssDNA overhangs, which are subsequently coated by replication protein A (RPA) [9, 10]. Displacement of RPA by Rad52 facilitates the assembly of the Rad51 nucleoprotein filament, which conducts homology search and strand invasion into a homologous donor sequence [11, 12]. While HR and NHEJ represent the primary repair mechanisms for DSB repair, cells can also invoke alternative pathways including single-strand annealing (SSA), microhomology-mediated end joining, break-induced replication (BIR), or *de novo* telomere addition (*dn*TA) [13–15]. Among these, BIR is particularly noteworthy for its ability to repair one-ended DSBs and collapsed replication forks. Although BIR typically depends on Rad51, a rare Rad51-independent variant of BIR remains insufficiently characterized [13, 16].

BIR is prominently observed in cells utilizing the alternative lengthening of telomere (ALT) pathway. In ALT cells, telomere elongation occurs independently of telomerase through mechanisms involving BIR, often utilizing subtelomeric regions as recombination substrates [17, 18]. The subtelomeric regions of yeast chromosomes are comprised of repetitive elements that facilitate recombination. All yeast subtelomeres contain an X element, followed on a subset of chromosome ends by one or more distal Y′ elements. In some cases, these repetitive elements are separated by interstitial telomeric sequences (ITSs) consisting of ∼80–150 bp of TG_1–3_ [2]. Significant progress has been made in understanding the types of events and genetic dependencies that support yeast ALT, but it remains a difficult phenomenon to study due to the ongoing instability generated in cells lacking telomerase [17–19].

In yeast, ITSs that perfectly match the telomeric repeat are found only within the subtelomeric sequences. However, telomere-like (TG-rich) sequences found throughout the genome can stimulate *dn*TA when exposed in ssDNA following a DSB [20]. Such sites of *dn*TA were first identified through studies of spontaneous rearrangements on the left arm of chromosome V [21–23]. Subsequently, additional sites have been identified both experimentally and computationally [24, 25]. Collectively, these sequences are named sites of repair-associated telomere addition (SiRTAs) for their ability to stimulate *dn*TA even when the initiating break occurs several kilobases distal to the site. Sequences predicted to function as SiRTAs are more common than expected by chance, with a particularly strong enrichment in subtelomeric regions where they may facilitate formation of a new ectopic telomere following a catastrophic telomere loss [25].

SiRTAs are bipartite: telomere addition predominantly occurs within a “Core” sequence, while a “Stim” sequence, located 5′ to the Core on the TG-rich strand, promotes *dn*TA. Multiple lines of evidence suggest that Stim sequences function through the recruitment of Cdc13, including direct binding assays *in vitro* [24]. SiRTA function requires 5′ end resection following a DSB to reveal Cdc13-binding sites in ssDNA, congruent with the observation that Cdc13 binding is detected at a SiRTA by chromatin immunoprecipitation only following induction of a break [24–26]. In addition, artificial recruitment of Cdc13 to a modified, inactive Stim sequence rescues *dn*TA [24]. Together, these experiments highlight Cdc13 as a key mediator of *dn*TA at SiRTAs. However, the potential involvement of Cdc13 in other DNA repair pathways remains insufficiently characterized, raising intriguing questions about its functional contributions to other DNA repair processes when bound to interstitial sites.

In an unpublished genetic screen designed to identify factors that stimulate *dn*TA at SiRTAs, we identified the ubiquitin protease, Ubp10. Loss of *UBP10* reduces *dn*TA frequency while concurrently increasing the frequency of other damage tolerance events at SiRTAs of various efficacies. We provide evidence that these effects are specific to SiRTAs and that a DNA sequence capable of binding Cdc13 is both necessary and sufficient (at least in some chromosome contexts) to promote translocations and/or large deletions in the absence of *UBP10*. When Ubp10 is lacking, SiRTAs utilize the repetitive subtelomeric regions of donor chromosomes to facilitate repair. Notably, some rearrangements at the SiRTA are independent of Rad51 and require Sir4 and Sir2 of the silent information regulator (SIR) complex, underscoring the diversity and complexity of DNA repair processes at these sites. This work reveals novel insights into the regulation of DNA repair mechanisms at interstitial Cdc13-binding sites and their implications for genomic stability.

## Materials and methods

### Yeast strains and plasmids

Strains were constructed in the S288C background as described [24–27] and are listed in Supplementary Table S1. Gene deletions were accomplished by one-step gene replacement using a selectable marker and verified by polymerase chain reaction (PCR) and sequencing [28]. Primer sequences are available in Supplementary Table S2.

Strain YKF2516 was created as follows. *URA3* was amplified from pRS305 [29] and integrated on the centromere-proximal boundary of SiRTA 6L-22 using primers 6URA3F and 6URA3R. An amplicon containing the Tel11 sequence (5′-GTGTGGGTGTG-3′) and flanking DNA was generated using overlap extension PCR with primers 6L22PreSiRTA For and 6L221Cdc13BS Rev (fragment I) and 6L221Cdc13BS For and 6L22PostSiRTA Rev (fragment II). Fragments I and II were extended by mutually primed synthesis using 6L-22 PreSiRTA For and 6L22 PostSiRTA Rev. The resulting product was transformed into 6L-22::*URA3* (YKF2509), cells were allowed to recover on rich medium for 24–48 h, then replica plated to medium containing 5-fluoroorotic acid (5-FOA). 5-FOA resistant (5-FOA^R^) isolates were screened by PCR and sequenced to confirm integration of the Tel11 sequence. Strain YKF2529 was made using the same technique to replace the Stim of SiRTA 9L-44 with two tandem copies of the Gal4 upstream activating sequence (2× Gal4 UAS).

Plasmid pRS314-*UBP10* was created by inserting a fragment encompassing the *UBP10* open reading frame (Chr. XIV, nucleotides 288 968–292 101) into pRS314 using BamHI and ApaI. Plasmid pRS314-*ubp10^C371S^* was created by generating an amplicon that included the desired mutation using primers C371S For and C371S Rev. The amplicon was inserted into pRS314 using BamHI and ApaI and sequenced to confirm insertion of the desired mutation.

### Inducible HO cleavage assay

The HO cleavage assay was performed as described [24–27, 30]. Briefly, cells were grown in synthetic complete media lacking uracil (SC-Ura) containing 2% raffinose to an OD_600_ of 0.6–0.8. Ten to thirty microliter aliquots were plated on yeast extract peptone medium containing 2% galactose (YEPG). Serially diluted cells were plated on rich medium containing 2% glucose (YEPD) to determine total viable cell count. For experiments requiring plasmid selection (pRS314), cells were plated on synthetic medium lacking leucine and containing either 2% glucose or 2% galactose. Plates were incubated at 30°C for 3 days. Surviving colonies were counted and ∼250 galactose-resistant (Gal^R^) colonies were patched onto plates containing 5-FOA medium to isolate GCR events (Gal^R^ 5-FOA^R^ colonies). Additional Gal^R^ 5-FOA^R^ colonies were identified by replica plating when necessary. The overall GCR frequency was calculated by multiplying the frequency of Gal^R^ colonies by the fraction of Gal^R^ colonies that survived on medium containing 5-FOA.

### Pooled telomere sequencing

Thirty Gal^R^ 5-FOA^R^ colonies, isolated as described earlier, were separately inoculated in 200 μl of liquid YEPD in a 96-well culture plate and incubated overnight at 30°C. Equal volumes (at least 40 μl) of each culture were pooled, and genomic DNA was extracted using the YeaStar genomic DNA kit (Zymo Research).

Libraries were prepared using 50 ng of genomic DNA and modified protocol using the Twist Library Preparation kit (Twist Bioscience 106 543) as described in [25]. DNA samples were randomly primed with 5′ barcoded adapters. Samples were pooled, captured on streptavidin-coated magnetic beads, and washed to remove excess reactants. A second 5′ adapter-tailed primer with a strand-displacing polymerase was used to convert the captured templates into dual adapter libraries. Beads were washed to remove excess reactants. Four cycles of PCR were performed to amplify the library and incorporate the plate barcode in the index read position. Libraries were sequenced using the NovaSeq 6000 with 150 bp paired-end reads, targeting 13–15 million reads per sample. Real Time Analysis software (version 2.4.11; Illumina) was used for base calling, and data quality control was completed using MultiQC v1.7. Library adapter sequences were removed using the Trimmomatic tool (Galaxy Version 0.38.0, [31]). Using Bowtie 2 sensitive local setting (Galaxy Version 2.5.0 + galaxy0), reads were identified that align to the region between the site at which the HO cut site was integrated and the last essential gene of each respective chromosome arm, referred to as the “repair region” [32, 33]. Using Bowtie 2 very fast setting (Galaxy Version 2.5.0 + galaxy0), these reads were realigned to the *Saccharomyces cerevisiae* S288C (sacCer3) reference genome and BAM files were used for subsequent analyses [34].

### Identification, characterization, and mapping of chromosomal rearrangement breakpoints

BAM files were uploaded to the Integrative Genomic Viewer, and reads were arranged by mapping quality [34]. “GCR split reads” were identified as reads where one end maps to the repair region and the other end maps elsewhere in the genome. GCR split reads were classified as deletions, translocations, or telomere addition events as follows. *Deletions*: Split reads contain nucleotides unambiguously aligning in unique sequence located telomere-proximal to the site of the *URA3* marker on the same chromosome as the HO cleavage site (HOcs). *Translocations*: Split reads contain nucleotides aligning to one or more genomic locations, excluding those defined as deletions. Split reads that could not unambiguously be classified as deletions were classified as translocations. *Telomere addition*: Split reads contain TG_1–3_ or C_1-3_A nucleotides (see examples in Supplementary Fig. S1A). The location of a GCR split read is defined as the genome coordinate at which the first nucleotide of the read diverges from the reference genome.

**Figure 1.**
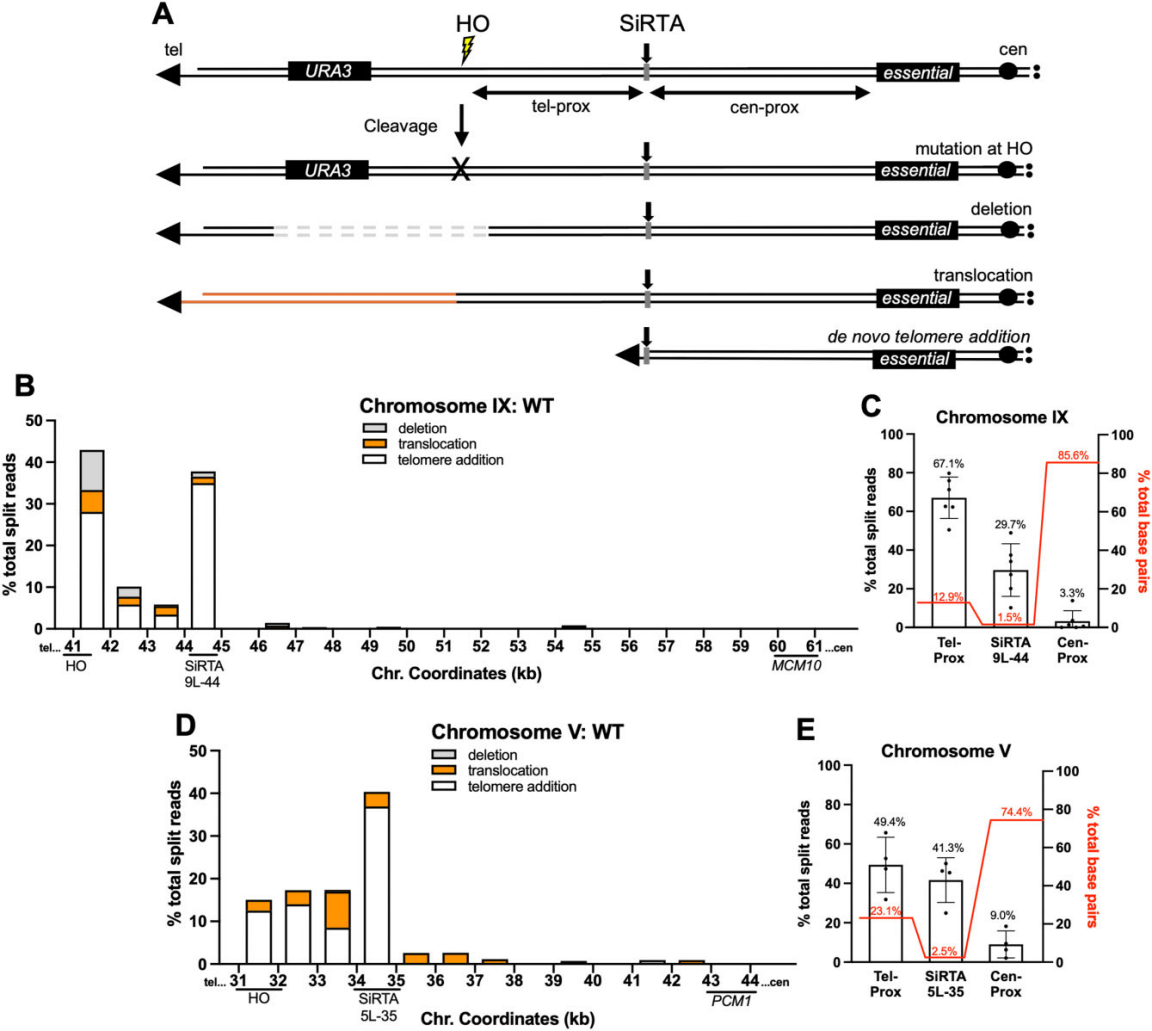
Gross chromosomal rearrangement (GCR) location and frequency are determined by mapping and quantifying split reads proximal to a site of induced cleavage. (**A**) Schematic diagram of the HO cleavage assay system. Cells that acquire resistance to galactose (used to induce expression of the HO endonuclease) and 5-FOA are assumed to have undergone a GCR event such as a deletion, translocation, or telomere addition. (**B**) Percentages of total split reads that map to each 1 kb interval within the repair region (HOcs to *MCM10*, most distal essential gene on chromosome IX) are shown. Split reads were classified as deletions (gray), translocations (orange), or telomere additions (white) as described in “Materials and methods” section. The *X*-axis is the distance (in kilobases) from the left end of the chromosome. SiRTA 9L-44 comprises 300 bp of the 44.0–45.0 kb region. Data are compiled from sequencing of 180 independent GCR events, analyzed in six pools of 30 clones each, as described in “Materials and methods” section. (**C**) The percentages of total split reads that map telomere-proximal to SiRTA 9L-44 (41 500–44 100), at SiRTA 9L-44 (44 100–44 400), and centromere-proximal to SiRTA 9L-44 (44 400–61 600) are shown (left *Y*-axis). Each data point represents a pooled sample of 30 clones that underwent a GCR event. Percentages of base pairs within the three specified regions are shown (red; right *Y-*axis). Percentages represent the fraction of base pairs in each region relative to the total base pairs across the entire repair region. (**D**) As in panel (B), with cleavage targeted to chromosome V. SiRTA 5L-35 comprises 300 bp of the 34.0–34.0 kb region. Translocation split reads observed in this region map distal to the SiRTA (see Supplementary Fig. S4A). *PCM1* is the most distal essential gene. Data are compiled from sequencing of 120 independent GCR events, analyzed in four pools. **(E)** As in panel (C), with split reads quantified telomere-proximal to SiRTA 5L-35 (31 900–34 700), at SiRTA 5L-35 (34 700–35 000), and centromere-proximal to SiRTA 5L-35 (35 000–44 000). Error bars denote standard deviation (SD).

Precise chromosome coordinates for each repair region analyzed are given in Fig. 1 and Supplementary Table S3. Previously published Nanopore sequencing data for the strains used in this study showed that the left arms of chromosomes IX, V, VI, and XIV closely resemble the S288C reference genome [26]. The total number of GCR split reads mapping to the repair region for each independent pool of 30 GCR events is recorded in Supplementary Table S4 (ranging from ∼100 to ∼550). Poisson distributions were generated to predict the number of reads expected per GCR event (Supplementary Fig. S1B). For experiments in which fewer than 200 GCR split reads occurred, rearrangements represented by one sequence read were included in the analysis to limit the possibility of eliminating true events. Single split reads were eliminated from experiments where >200 GCR split reads occurred given the low likelihood that these represent true events. Following this adjustment, the frequency of GCR split reads at a given location and/or of a given type was quantified by dividing the number of GCR split reads meeting those criteria by the total number of GCR split reads mapping to the repair region.

## Results

### Mapping and quantification of rearrangement breakpoints following a DSB

In this work, GCR formation is monitored in haploid cells using an established assay in which a single recognition site for the HO endonuclease is integrated distal to the last essential gene on a chromosome of interest (Fig. 1A). The HO endonuclease is placed under control of a galactose-inducible promoter. Cells that survive persistent nuclease expression repair the break in a way that prevents further cleavage, either through point mutations or indels within the HOcs or through GCR events (large deletions, translocations, or telomere additions) that completely eliminate the cleavage site. By selecting for loss of a *URA3* marker integrated distal to the HOcs, we identify those clones in which a GCR event occurred between the HOcs and the most distal essential gene on that chromosome arm, here referred to as the “repair region” (Fig. 1A). In previous work, we used this assay to demonstrate that SiRTAs are hot spots for telomere addition, even when located at least 3 kb proximal to the cleavage site [24–26, 35].

Previously, we validated a pooled telomere sequencing approach to quantify the frequency of telomere addition events at the SiRTA [25]. In brief, genomic DNA is isolated from a pool of 30 clones that survived nuclease induction on galactose, accompanied by loss of *URA3* expression (Gal^R^ 5-FOA^R^). This pooled DNA is subjected to deep, short-read (150 bp) sequencing. Among sequence reads aligning to the SiRTA, those showing evidence of a telomere addition (TG_1–3_ or C_1–3_A sequence) are counted. Read depth is normalized between experiments using read count at the most distal essential gene. This method yields results that correlate strongly with data obtained by PCR-based mapping (*r*^2^ = 0.97), allowing accurate estimation of the fraction of surviving cells undergoing telomere addition at the SiRTA. Our original analyses did not attempt to detect or quantify other types of rearrangements either at the SiRTA or elsewhere within the region from the HOcs to the last essential gene.

To comprehensively identify rearrangement events, we modified the analysis by incorporating the Integrative Genomic Viewer software. Using the *S. cerevisiae* S288C reference genome (sacCer3), reads are aligned to the “repair region” extending from the site at which the HOcs was integrated to the distal boundary of the last essential gene (representing the most extensive chromosome loss event compatible with cell viability) [34]. Aligned reads are arranged by mapping quality; reads with low mapping quality are predominantly split reads where the proximal portion of the read aligns to the region of interest and the distal portion aligns elsewhere in the reference genome. Split reads containing TG_1–3_ or C_1–3_A nucleotides are classified as telomere addition events (Supplementary Fig. S1A). Split reads containing nucleotides unambiguously mapping to single-copy sequences distal to the *URA3* marker on the same chromosome arm are classified as deletions. Split reads containing nucleotides unambiguously mapping to locations on other chromosomes are classified as translocations. In some cases, the split read contains sequences that map to multiple locations (typically in subtelomeric repeats). These events are classified as translocations, although some may be large deletions if the distal sequence involved in the rearrangement event derives from the same chromosome as the induced DSB. Such events are discussed in more detail below. Because the 30 clones included in the pool have all lost the end of the cleaved chromosome (as defined by loss of *URA3*), we use the percentage of total split reads at a particular nucleotide or region as a proxy for the percentage of total GCR events at that location. This approach allows us to determine the nature and frequency of chromosomal rearrangements and to map the location of each event with nucleotide precision (Fig. 1B and D; see “Materials and methods” section for details).

We applied this methodology to comprehensively map GCR events in the vicinity of two sequences previously shown to function as SiRTAs. SiRTA 9L-44 (∼44.0 kb from the endogenous telomere on the left arm of chromosome IX) lies 2.7 kb upstream of the HOcs and 17.2 kb distal to the last essential gene, *MCM10*. SiRTA 5L-35 (∼35 kb from the endogenous telomere on the left arm of chromosome V) lies 2.8 kb upstream of the HOcs and 9.0 kb from the last essential gene, *PCM1*. The “repair region” includes ∼19.9 kb on chromosome IX (HOcs to *MCM10)* and ∼12.1 kb on chromosome V (HOcs to *PCM1*). On chromosome IX, we combined data from six experiments representing a total of 180 independent GCR events. Sequence reads were aligned to the repair region and all split reads were identified and quantified. SiRTA 9L-44 comprises 1.5% of the entire repair region, yet 29.7% of total split reads map to the SiRTA (Fig. 1C). Of the split reads mapping precisely to SiRTA 9L-44, 94.5% contain telomeric repeats, while the remaining 6.5% contain nucleotides indicative of a translocation/deletion (Supplementary Fig. S2A).

Similar results were obtained for SiRTA 5L-35. Combining results from four independent experiments (120 GCR events), 41.3% of total split reads map to the SiRTA itself (Fig. 1E). SiRTA 5L-35 comprises 2.5% of the repair region on chromosome V. All split reads that map to SiRTA 5L-35 contain telomeric repeats (Supplementary Fig. S2B). We previously found that deletion of *RAD52* does not alter the frequency of telomere addition at SiRTAs 9L-44 and 5L-35, consistent with acquisition of telomeric sequences by telomerase rather than by recombination with telomeric or subtelomeric sequences (an event expected to require *RAD52*) [26]. Therefore, at SiRTAs 9L-44 and 5L-35, we make the simplifying assumption that all events showing acquisition of TG_1–3_ sequences represent addition of a new telomere by telomerase (i.e. they are *dn*TA events).

This methodology is robust and reproducible. At SiRTA 9L-44, calculating the frequency of GCR events at the SiRTA using the percentage of total split reads that map to that sequence gives values statistically indistinguishable from values obtained using methods that normalize to total read depth or that quantify individual events by PCR (Supplementary Fig. S2C). We previously observed that deletion of *RAD51* increases the GCR events centromere-proximal to SiRTA 9L-44 [26]. Using this approach, we observed statistically indistinguishable values to those previously reported, not only at the SiRTA, but across the entire repair region (Supplementary Fig. S2D). Given this congruence, we use the terms “fraction of split reads” and “fraction of GCR events” interchangeably throughout this work. Importantly, GCR split reads are exclusively observed on the chromosome arm where the break is induced. Analysis of the same region on chromosome IX when the cleavage is induced on chromosome V shows no evidence of GCR split reads suggesting that GCR split reads are a result of cleavage (Supplementary Table S5).

### The deubiquitinase activity of Upb10 suppresses translocations at SiRTAs

**Figure 2.**
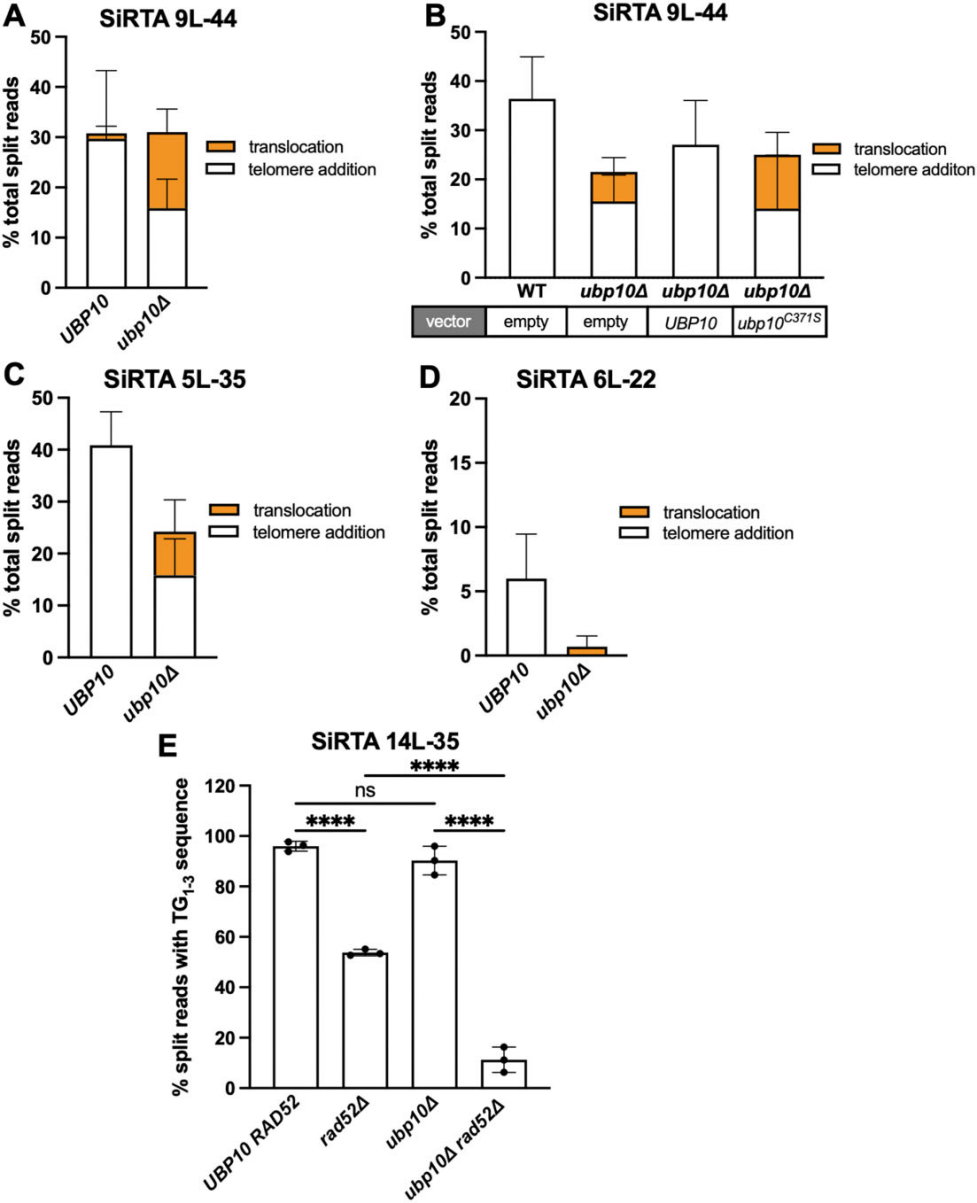
Loss of *UBP10* increases the frequency of translocations at SiRTAs. Percentages of total split reads mapping to the indicated SiRTA are shown for *UBP10* and *ubp10Δ* strains. Chromosome coordinates for each SiRTA are given in Supplementary Table S3. Split reads were classified as translocations (orange) or telomere additions (white) as described in “Materials and methods” section. No deletions were observed. (**A**) Data for SiRTA 9L-44 are from six pools of 30 GCR events each (*UBP10* samples are the same as those described in Fig. 1B and C). (**B**) *UBP10* and *upb10Δ* strains were transformed with either empty vector or a plasmid expressing a WT or catalytically dead variant of *UBP10* as indicated. Data are from three pools of 30 GCR events for each strain. (**C**) Data for SiRTA 5L-35 are from four pools of 30 GCR events each (*UBP10* samples are the same as those described in Fig. 1D and E). (**D**) Data for SiRTA 6L-22 are from three pools of 30 GCR events each. (**E**) Percentages of total split reads mapping to SiRTA 14L-35 are shown for the indicated strains. Data are from three pools of 30 GCR events each. ****P < .0001, analysis of variance (ANOVA) with Tukey’s multiple comparison test. Error bars denote SD.

In an unpublished screen to identify trans*-*acting factors that promote *dn*TA, we identified the ubiquitin protease, Ubp10. The consequence of deleting *UBP10* was initially measured at SiRTA 9L-44. Deletion of *UBP10* does not change the fraction of total GCR events at the SiRTA, but there is a dramatic shift from *dn*TA to translocations, with translocations accounting for nearly half of the GCR events at the SiRTA (Fig. 2A and Supplementary Fig. S3A–C). Expressing *UBP10* from its endogenous promoter on a low-copy number, centromere-containing plasmid in the *ubp10Δ* strain restores *dn*TA and eliminates translocations (Fig. 2B). In contrast, expression of the catalytically dead *ubp10^C371S^* allele fails to complement the mutant defect (Fig. 2B), suggesting that suppression of translocations requires the deubiquitinase activity of Ubp10.

To determine the generalizability of this effect, we examined the impact of deleting *UBP10* at three additional SiRTAs that differ markedly in the frequency of *dnTA*. At SiRTA 5L-35, the fraction of GCR events mapping to the SiRTA decreases upon *UBP10* deletion (Fig. 2C). Translocations at SiRTA 5L-35 increase (from none in the *UBP10* strain to 25.5% in the *ubp10Δ* background), but the effect is less pronounced than at SiRTA 9L-44 (compare Fig. 2C to Fig. 2A; Supplementary Fig. S3D–F). SiRTA 6L-22 (left arm of chromosome VI) stimulates frequencies of *dn*TA that marginally meet the threshold to be considered a functional SiRTA [defined as 6.6% [25, 30], Supplementary Fig. S3G]. Upon deletion of *UBP10*, telomere addition events are eliminated (Fig. 2D and Supplementary Fig. S3H). Translocation split reads were observed in two of three experiments at a frequency suggesting a single translocation event of 90 GCR clones analyzed (Fig. 2D and Supplementary Fig. S3I), indicating that SiRTA 6L-22 only weakly stimulates any type of GCR event.

At the other end of the spectrum, we examined a sequence found ∼35 kb from the endogenous telomere on the left arm of chromosome XIV (SiRTA 14L-35) that stimulates high levels of telomere addition (∼95% of all GCR events) (Fig. 2E and Supplementary Fig. S3J). We detected no apparent difference in the frequency of telomere addition events and no translocations upon deletion of *UBP10* (Fig. 2E). However, because the sequence of SiRTA 14L-35 bears high similarity to a telomeric repeat, we suspected that at least a fraction of telomere addition events assumed to arise through telomerase action might instead be translocations to subtelomeric or telomeric regions. Consistent with this hypothesis, we observe a significant (1.8-fold) decrease in the frequency of split reads at the SiRTA when *RAD52* is deleted (Fig. 2E). We infer that, in contrast to our prior observations at SiRTAs 5L-35 and 9L-44 [26], nearly half of the telomere addition events at SiRTA 14L-35 occur through recombination with existing telomeric repeats. In the *ubp10Δ* background, deletion of *RAD52* causes an even more pronounced (6.9-fold) reduction in telomere addition (Fig. 2E). Because the fraction of *RAD52*-dependent events (i.e. translocations) increases upon deletion of *UBP10*, we conclude that Ubp10 suppresses telomeric translocations at SiRTA 14L-35. Taken together, these results suggest that the deubiquitylation of one or more substrates by Ubp10 promotes *dn*TA by telomerase and represses formation of translocations at SiRTAs.

### Suppression of genome rearrangements by *UBP10* is most pronounced at SiRTAs

**Figure 3.**
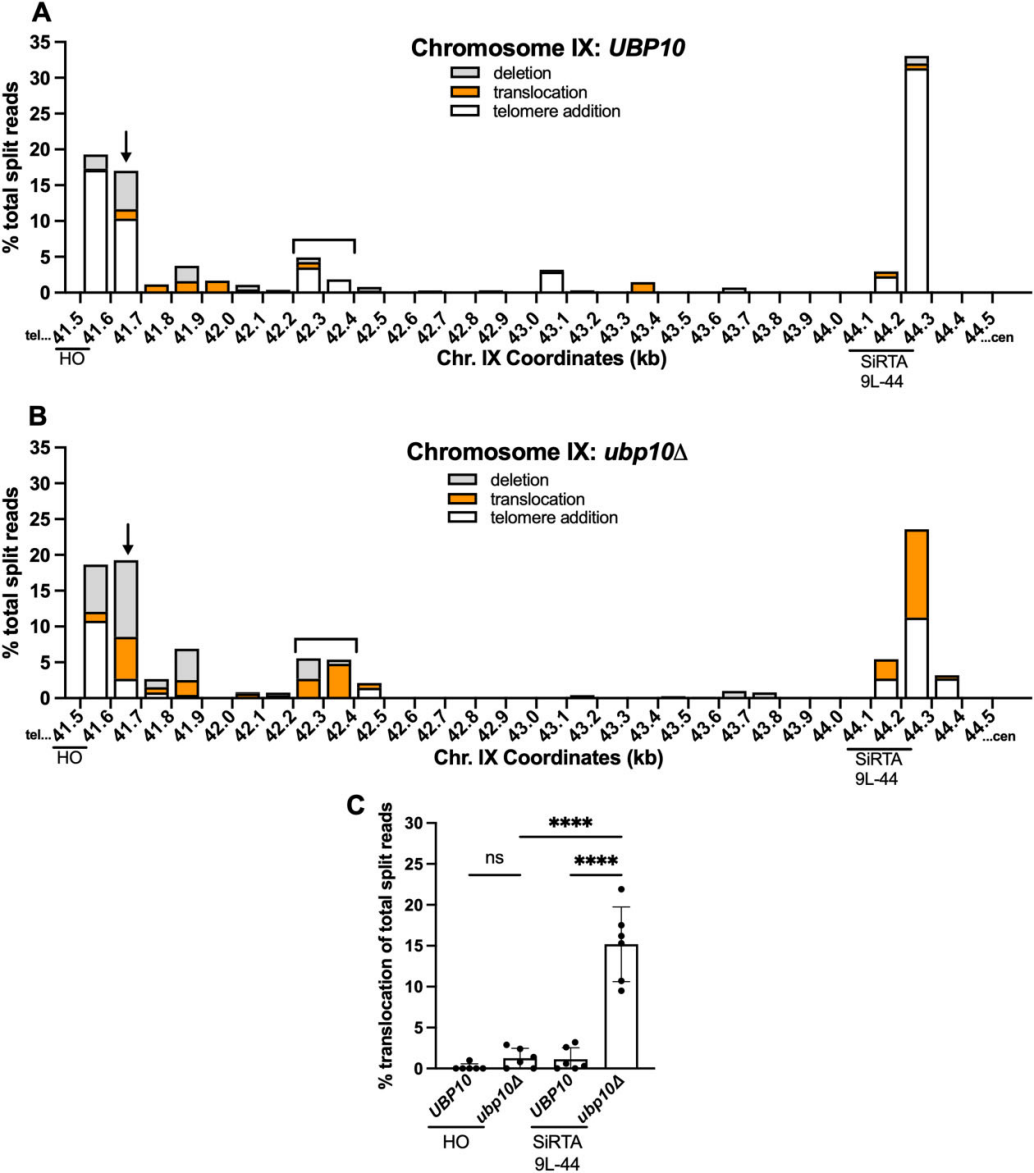
The effects of Ubp10 are most pronounced at SiRTAs. (**A, B**) Percentages of total split reads that map to each 100 bp interval between the HOcs and SiRTA 9L-44 are shown. Split reads were classified as deletions (gray), translocations (orange), or telomere additions (white) as described in “Materials and methods” section. The *X*-axis is the distance (in kilobases) from the left end of the chromosome. Data for *UBP10* and *ubp10Δ* strains are the same as described in Fig. 2A. The arrow and brackets indicate regions where *dn*TA decreases and translocations/deletions increase upon loss of *UBP10* (see text and Supplementary Fig. S5A–C). **(C)** Translocation split reads that map within the 100 bp region telomere proximal to the HOcs (41.5–41.6 kb) or to SiRTA 9L-44 are quantified. Percentages are calculated relative to total split reads mapping to the repair region, from the HOcs to the last essential gene. Each data point corresponds to one of the six pools of 30 GCR events as described in Fig. 2A. ****P < .0001, ANOVA with Tukey’s multiple comparisons test. Error bars denote SD.

To determine whether the effects of *UBP10* deletion are specific to SiRTAs, we analyzed the types and locations of GCR events within and distal to SiRTA 9L-44 (accounting for 96.8% of events arising after cleavage on chromosome IX; Fig. 1C) in the presence and absence of *UBP10* (compare Fig. 3A and B). The overall pattern of events is similar in the two strains, with no evidence of new rearrangement hotspots upon deletion of *UBP10*. This observation holds true for the region between the HOcs and SiRTA 5L-35 on chromosome V (Supplementary Fig. 4A and B), suggesting that Ubp10 affects repair pathway choice at sequences already prone to mutational repair.

In addition to the previously identified SiRTA 9L-44, telomere addition is favored (>5% of total split reads) at two additional sites in the *UBP10* strain. The first site is 200 bp proximal to the HOcs (41.6–41.7 kb), where >15% of GCR events occur (Fig. 3A and B, arrows). In this region, the fraction of events involving telomere addition is strongly reduced by deletion of *UBP10*, with concomitant increase in translocations and deletions (Fig. 3A and B; Supplementary Fig. S5A). The second site is between 42.2 and 42.4 kb from the endogenous telomere (Fig. 3A and B, brackets). This site undergoes telomere addition in the *UBP10* strain but incurs translocations and deletions in the absence of *UBP10* (Fig. 3A and B; Supplementary Fig. S5B). To determine if these regions contain a TG-rich sequence similar to SiRTA 9L-44, we calculated the percentage of total nucleotides that are either T or G (T + G) and the ratio of G to T nucleotides in a sliding window along the strand running 3′–5′ between the HOcs and SiRTA 9L-44. In this region, SiRTA 9L-44 has the highest G/T ratio, but the 41.6–41.7 kb and 42.2–42.4 kb regions also have elevated G/T ratios compared to their surrounding sequences (Supplementary Fig. S5C). This analysis suggests that these telomere-like sequences may function similarly to SiRTAs.

Deletion of *UBP10* does not increase the frequency of translocations under all circumstances. The HOcs itself is a “hotspot” of *dn*TA, at least in part because cleavage generates a TGTT 3′ overhang upon which telomerase may act (Supplementary Table S3). We compared the frequency of translocation split reads at the HOcs (41.5–41.6 kb) and at SiRTA 9L-44 in the presence or absence of *UBP10*. In contrast to the striking increase in translocation events observed at SiRTA 9L-44, translocations do not significantly increase at the HOcs in the absence of *UBP10* (Fig. 3C). The same result is observed on chromosome V (Supplementary Fig. S4C). Telomerase action at the HOcs is likely stimulated by the Ku heterodimer (Yku70/80), which associates with the DNA end to recruit telomerase through interaction with the telomerase RNA [36]. These events appear unaffected by deletion of *UBP10*.

In summary, the patterns of GCR events incurred following a DSB on chromosome IX (Fig. 3 and Supplementary Fig. S5) and V (Supplementary Fig. S4) suggest that Ubp10 preferentially suppresses translocations at sites that are hotspots of *dn*TA, most prominently those at which *dn*TA is stimulated by the association of Cdc13 with ssDNA following a DSB [24–26]. These observations suggest that Cdc13 may stimulate chromosome rearrangements in the absence of *UBP10*, a possibility addressed in the next section.

### The SiRTA stim sequence is required to stimulate rearrangements in the absence of *UBP10*

**Figure 4.**
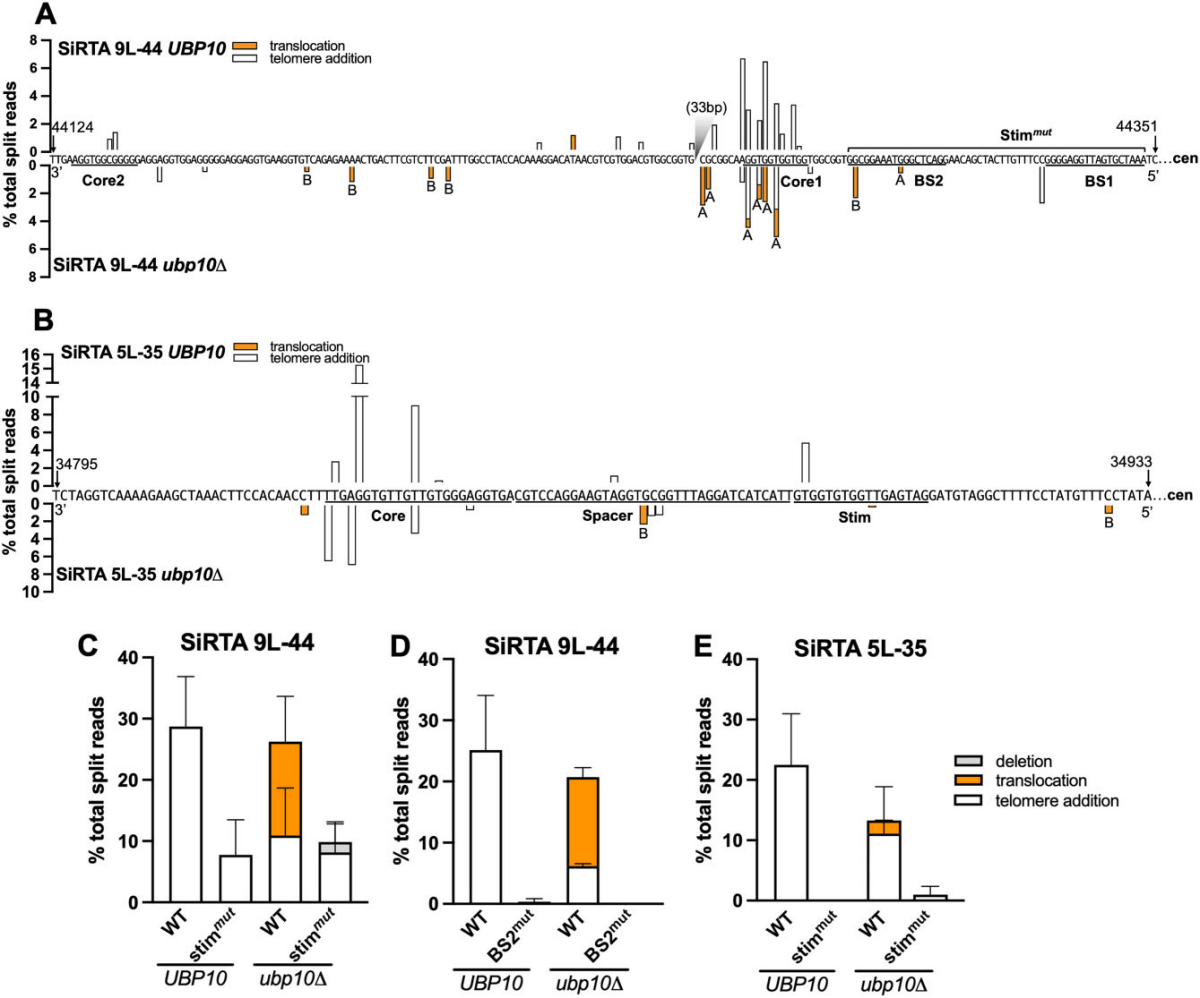
Mutation of the SiRTA Stim sequence eliminates rearrangements in the absence of *UBP10*. Nucleotide-resolution maps showing sites of telomere additions (white) or translocations (orange) at SiRTA 9L-44 (**A**) or SiRTA 5L-35 (**B**) using the data summarized in Fig. 2A and C. Sequences are oriented 3′–5′ with the centromere located to the right (nucleotide coordinates are indicated). Percentages are calculated relative to total split reads mapping from the HO site to the last essential gene. Bars above the sequence correspond to the *UBP10* strain; bars below the sequence correspond to the *ubp10Δ* strain. In panel (A), a 33 bp region between Core 1 and Core 2 that is devoid of events is indicated with a gray triangle. (**C**) Percentages of total split reads mapping to SiRTA 9L-44 or a mutated SiRTA 9L-44 (Stim^mut^) are shown in *UBP10* and *ubp10Δ* strains. To generate SiRTA 9L-44 Stim^mut^, the 52-bp Stim region of 9L-44 was replaced with a non-telomeric sequence. Data are from three pools of 30 GCR events. (**D**) Percentages of total split reads mapping to WT SiRTA 9L-44 or a mutated SiRTA 9L-44 (BS2^mut^) are shown in *UBP10* and *ubp10Δ* strains. To generate 9L-44 BS2^mut^, the 18 bp that compose SiRTA 9L-44 Cdc13 binding site 2 (BS2) were replaced with polyadenine. Data are from 2 pools of 30 GCR events. (**E**) Percentages of total split reads mapping to WT SiRTA 5L-35 or a mutated SiRTA 5L-35 (Stim^mut^) are shown in *UBP10* and *ubp10Δ* strains. To generate 5L-35 Stim^mut^, the 18-bp Stim region of SiRTA 5L-35 was replaced with polyadenine. Data are from three pools of 30 GCR events. Error bars denote SD.

We previously determined that SiRTAs 9L-44 and 5L-35 comprise a bipartite structure in which telomere addition at one or more Core sequences is promoted by a region (the “Stim”) located more distal to the chromosome break [24]. Multiple lines of evidence suggest that the Stim sequence associates with Cdc13 following resection of a distal DSB [24, 25]. We first examined how deletion of *UBP10* affects the precise location of *dn*TA and translocation events in the SiRTA. As previously reported, the majority of GCR events at SiRTA 9L-44 in the *UBP10* background involve telomere addition within the TG-rich Core 1 region, with a fraction of events occurring at Core 2, located ∼100 bp distal to Core 1 (Fig. 4A) [24]. With improved resolution, it is apparent that some telomere addition events occur between Core 1 and Core 2. In the absence of *UBP10, dn*TA events are reduced in frequency but continue to cluster in Core 1. Translocation breakpoints predominantly occur in and near Core 1 (Fig. 4A). At SiRTA 5L-35, *dn*TA events are clustered in the Core sequence in the *UBP10* strain, as previously reported [24]. Upon deletion of *UBP10*, the overall pattern of *dn*TA is unchanged. In contrast to SiRTA 9L-44, translocations at SiRTA 5L-35 are not favored at the Core sequence, with a fraction mapping proximal to the previously defined Stim sequence (Fig. 4B).

Mutation of the Stim regions of SiRTAs 5L-35 or 9L-44 strongly reduces the frequency of *dn*TA by presumably eliminating or reducing Cdc13 association [24]. To test whether translocations observed in the absence of *UBP10* require a Cdc13 binding site, we replaced the entire Stim region of SiRTA 9L-44 (consisting of two Cdc13 binding sites, BS1 and BS2; Fig. 4A) with a non-telomeric sequence (Stim^mut^). Importantly, this mutation does not alter sequences that engage in the majority of *dn*TA or translocation events (Fig. 4A). As expected, Stim^mut^ significantly reduces *dn*TA in the *UBP10* strain (Fig. 4C and Supplementary Fig. S6A). Strikingly, translocations previously observed in the *ubp10Δ* background are eliminated by Stim^mut^ (Fig. 4C and Supplementary Fig. S6B). To verify this result, we mutated BS2 only, previously shown to eliminate *dn*TA at SiRTA 9L-44 [24]. Indeed, mutation of BS2 eliminates translocations and *dn*TA events in the *ubp10Δ* strain (Fig. 4D). It is unclear why the BS2 mutation has a stronger phenotype than Stim^mut^, but the difference may arise from sequences used to replace the Stim in each case. Mutating the Stim sequence at SiRTA 5L-35 in the *ubp10Δ* background eliminates translocations, although significance is difficult to ascertain since translocation frequencies are low at SiRTA 5L-35 (Fig. 4E). Taken together, these results show that the Stim sequence is required to promote translocations at SiRTAs in the absence of *UBP10*, implicating Cdc13 in the stimulation of both *dn*TA and translocations.

**Figure 5.**
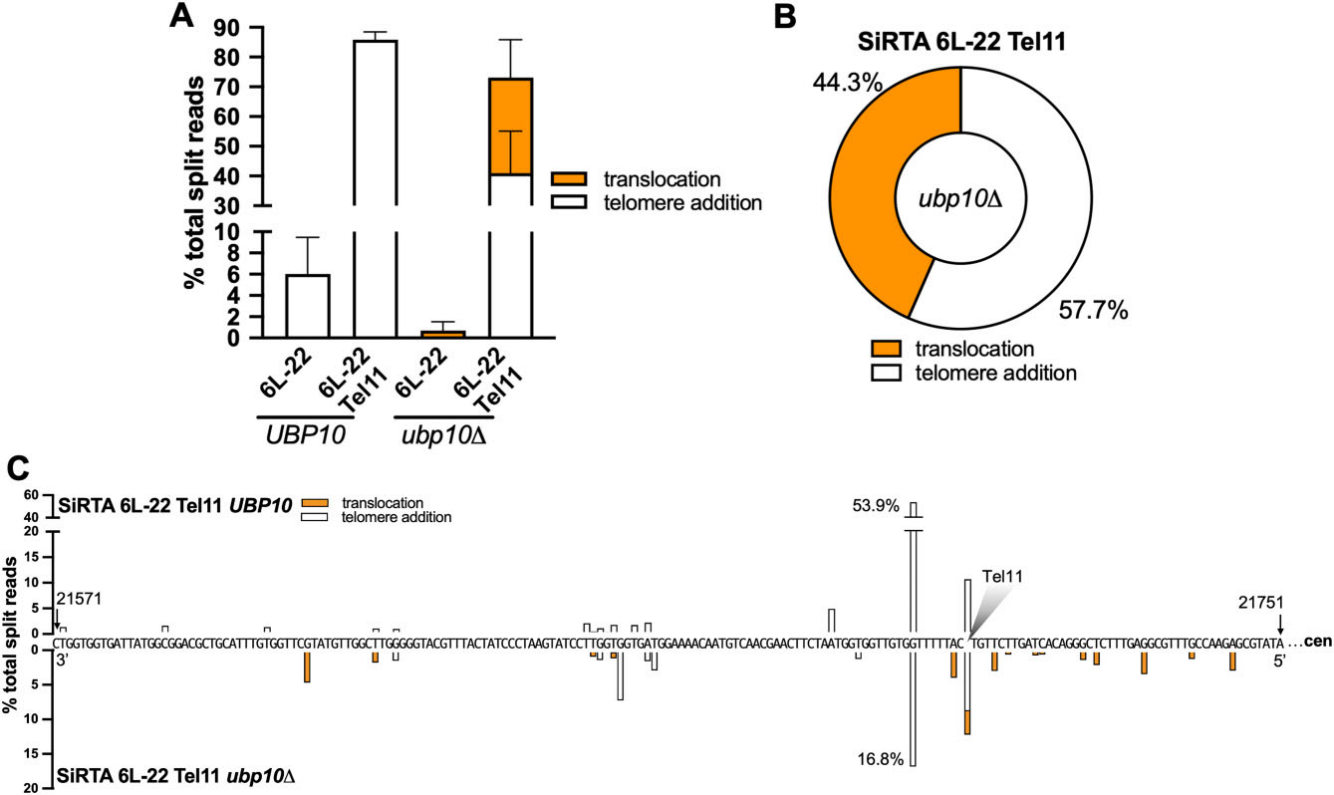
Integration of a single Cdc13 binding site is sufficient to stimulate translocations in the absence of *UBP10*. (A–C) DNA repair events were monitored across the region containing SiRTA 6L-22 and SiRTA 6L-22 Tel11 in *UBP10* and *ubp10Δ* strains. To generate SiRTA 6L-22 Tel11, the Tel11 sequence (5′-GTGTGGGTGTG-3′) was integrated upstream of SiRTA 6L-22. Data are from three pools of 30 GCR events each in each strain. (**A**) Percentages of total split reads that map to SiRTA 6L-22 or SiRTA 6L-22 Tel11 are shown. Data for SiRTA 6L-22 are repeated from Fig. 2D for comparison. (**B**) Among split reads mapping to SiRTA 6L-22 Tel11, the percentages that involve telomere additions (white) or translocations (orange) are shown for the *ubp10Δ* strain. (**C**) Nucleotide-resolution maps showing sites of *dn*TA and/or translocations at SiRTA 6L-22 Tel11. The sequence is oriented 3′–5′ with the centromere located to the right (nucleotide coordinates are indicated). Bars above the sequence correspond to the *UBP10* strain; bars below the sequence correspond to the *ubp10Δ* strain. The shaded triangle represented the site at which the Tel11 sequence was integrated.

If exposure of a Cdc13 binding site within the resected strand following a DSB is required to stimulate both *dn*TA and translocations (in the absence of *UBP10*), we reasoned that addition of a recognition site for Cdc13 to the extremely weak SiRTA 6L-22 might be sufficient to increase both types of events. *In vitro*, Cdc13 binds an 11-base ssDNA oligonucleotide representative of yeast telomeric DNA sequence (Tel11; 5′-GTGTGGGTGTG-3′) with 3pM affinity [37]. Integration of Tel11 upstream of the weakly functional SiRTA 6L-22 sequence dramatically increases the frequency of *dn*TA events at the SiRTA in the *UBP10* strain (from ∼6% to ∼85%; Fig. 5A, Supplementary Fig. S7A and B). Consistent with Tel11 serving as a Stim sequence, most *dn*TA events occur distal to Tel11 (Fig. 5C). While the frequency of GCR events is unaffected by deletion of *UBP10*, repair shifts from *dn*TA to translocations, with translocations accounting for 44% of the total events at the mutated SiRTA (Fig. 5A and B; Supplementary Fig. S7C). Similar to the situation at SiRTA 5L-35, we observe translocations upstream of the Tel11 sequence, suggesting that Cdc13, bound to the ssDNA produced by resection, can facilitate translocations even when the binding site is excluded from the product of the repair event (Figs 4B and 5C). Taken together, these data strongly argue that a Cdc13 binding site is necessary and sufficient to stimulate translocations at SiRTAs in the absence of *UBP10*.

### Translocations at SiRTAs primarily target subtelomeric sequences

Split reads contain not only the site within the SiRTA at which a translocation occurs, but also the sequence to which the SiRTA is joined (denoted here as the “target” sequence). The vast majority of translocations involve target sequences within the subtelomeric X or Y′ elements, either on the same chromosome arm as the break (in *cis*) or on a different chromosome arm (in *trans*). Subtelomeric translocations can be further subdivided into three classes (A–C) as described below.

**Figure 6.**
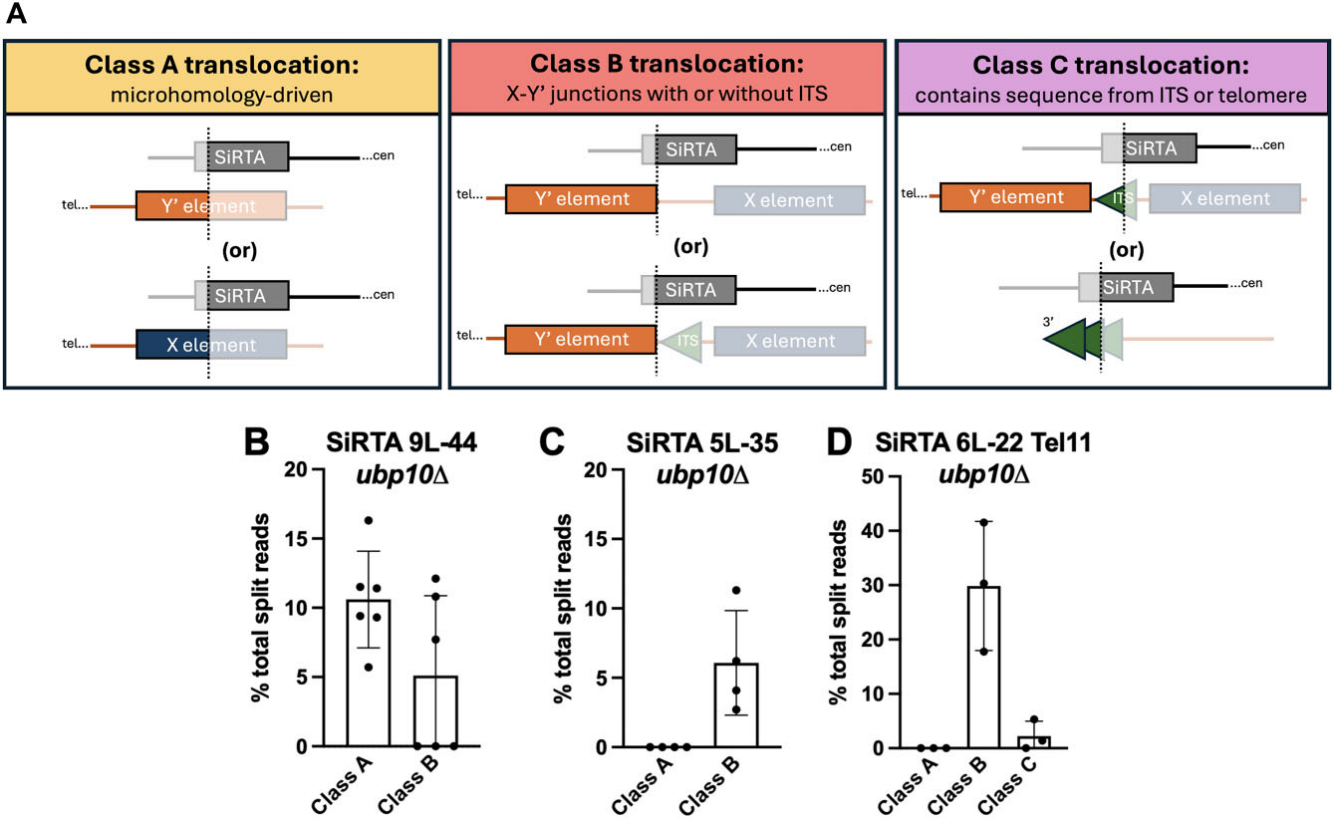
Ubp10 suppresses subtelomeric recombination at SiRTAs. (**A**) Schematic diagram depicting the classes of translocation breakpoints observed between the SiRTA (top) and the putative subtelomeric region (bottom). Diagrams are oriented with the centromere to the right. Saturated colors illustrate the product observed in the translocation split read. The dotted line represents the translocation breakpoint. (**B**–**D**) Translocation split reads previously mapped to SiRTA 9L-44 (Fig. 2A), SiRTA 5L-35 (Fig. 2C), or SiRTA 6L-22 Tel11 (Fig. 5A) were classified as Class A, Class B, or Class C and quantified as the percentage of total split reads between the HOcs and the last essential gene. Each data point is generated from a pool of 30 GCR events. At SiRTA 5L-35, a fraction of translocations at the SiRTA is neither Class A nor B, with breakpoints in non-telomeric regions (average of 2% of total split reads).

Class A translocations, observed thus far only at SiRTA 9L-44, join the SiRTA to a site internal to either an X element or a Y′ element (Fig. 6A, Supplementary Figs 8 and 9A, and Supplementary Table S7). For these split reads, the sequence distal to the breakpoint shows high nucleotide similarity to 10 potential subtelomeric targets, with no other matches in the genome. Although X and Y′ elements show high levels of sequence identity, the Class A split reads are identical only to X and Y′ elements on chromosomes IX-L and X-L; all other potential subtelomeric targets differ by at least three nucleotides (Supplementary Table S7). The strain utilized in this work is from the S288C background, and our prior analysis is consistent with high similarity to the reference strain [26]. Therefore, Class A “translocations” are either large, internal deletions that join the SiRTA with the *X* or *Y*′ element on the left arm of chromosome IX (*cis*) or are true translocations that join the SiRTA with either the X or Y′ element on the left arm of chromosome X (*trans)*. Class A breakpoints occur at several closely clustered sequences at SiRTA 9L-44 (near Core 1; Fig. 4A). Examination of sequences flanking the breakpoint at the SiRTA and the subtelomeric target sequence reveal, in each case, a region of interrupted microhomology (19–33 bp) (Supplementary Fig. S9A). The restriction of Class A events to SiRTA 9L-44 likely reflects the existence of fortuitous microhomology, perhaps combined with a higher likelihood of repair occurring in *cis* to the original DSB.

Class B translocations join the SiRTA to a sequence found at the beginning of a subset of full-length Y′ elements (Fig. 6A, Supplementary Figs S8 and S9B, and Supplementary Table S8). This is the most ubiquitous class of translocations, occurring at SiRTAs 9L-44, 5L-35, and the modified 6L-22 Tel11 (Fig. 6B–D). The characteristic 5′-ATATATAT-3′ sequence at the breakpoint of the Class B translocations is found 12 times at subtelomeres, but the sequence located immediately proximal to this sequence motif varies (Supplementary Fig. S9B). In seven cases, the ATATATAT motif is immediately preceded by an ITS, while in the other five cases, the preceding sequence is unique or nearly unique (Supplementary Table S8). The subtelomeres of V-L and IX-L do not contain the ATATATAT motif, so the events observed at SiRTAs 5L-35 and 9L-44 must be translocations. The motif does occur on chromosome VI-L, so it is possible that at least some events at SiRTA 6L-22 Tel11 occur in *cis*.

Given the read length and high level of homology between X and Y′ elements, we cannot determine which X and Y′ elements are targeted (Supplementary Fig. S9B). However, there is no obvious sequence similarity between the SiRTA sequences that are involved in Class B translocations and little or no microhomology that could explain the exquisite targeting of these translocations to the first nucleotide of these particular Y′ elements (Supplementary Fig. S9B). For this reason, we favor the hypothesis that Class B translocations occur at one of the seven Y′ elements that are preceded by an ITS. Although SiRTAs contain telomere-like sequences, the 20 bp preceding the breakpoints of the Class B translocations are not remarkably TG-rich (Supplementary Fig. S9B), reinforcing the conclusion that Class B translocations are not microhomology mediated.

Class C translocations occur at SiRTA 14L-35 and are described above as events that resemble *dn*TA but require *RAD52* (Fig. 3D and Fig. 6A), suggesting a homology-mediated recombination event between SiRTA 14L-35 and either terminal or interstitial telomeric repeats. A few Class C translocation split reads show evidence of Y′ sequence following the telomeric tract, consistent with at least a fraction of translocations occurring at an ITS (Supplementary Fig. S9C). Interestingly, although not perfectly homologous, the highest scoring match is the ITS on the left arm of chromosome XIV, consistent with a large deletion. Due to limitations of short-read sequencing, we are unable to quantify the relative use of the interstitial versus the terminal tract as a repair template. Our prior observation that deletion of *RAD52* fails to reduce telomere addition at SiRTAs 9L-44 or 5L-35 [26] suggests that Class C translocations are mostly limited to sites, like SiRTA 14L-35, that contain a long tract of sequence with high similarity to the telomeric TG_1–3_ pattern.

We observed two events at SiRTA 6L-22 Tel11 in which the ATATATAT motif is immediately preceded by TG-rich sequence that is not present at the SiRTA. In one case, the preceding sequence is 33 nucleotides of TG_1–3_ sequence, consistent with a Class C event (Supplementary Fig. S9C). Although imperfect, the closest match to this sequence is the ITS on the left arm of chromosome VI, suggesting again a preference for interactions in *cis* to the break. The other event contains “GTGT” and may represent a Class C event (there are two sites where this sequence precedes the ATATATAT motif). Alternatively, we cannot rule out the possibility that telomerase extended the 3′ end at the SiRTA as an intermediate step in repair.

At SiRTA 5L-35, ∼2% of total GCR events were translocation split reads that joined chromosome V to a non-telomeric sequence of a different chromosome arm. Some target sequences are located >100 kb from the nearest telomere and oriented in a way that would produce a dicentric chromosome. These breakpoints likely represent the initiation of a more complex rearrangement. It is unclear why this type of translocation is preferentially observed at SiRTA 5L-35. These non-subtelomeric translocations are not observed in the SiRTA 5L-35 Stim^mut^  *ubp10Δ* strain, suggesting that they depend on Cdc13 association. A DNA sequence capable of Cdc13 binding is not sufficient, however, since no events of this type are observed at SiRTA 6L-22 Tel11.

### Ubp10 represses homologous recombination repair mechanisms at SiRTAs

**Figure 7.**
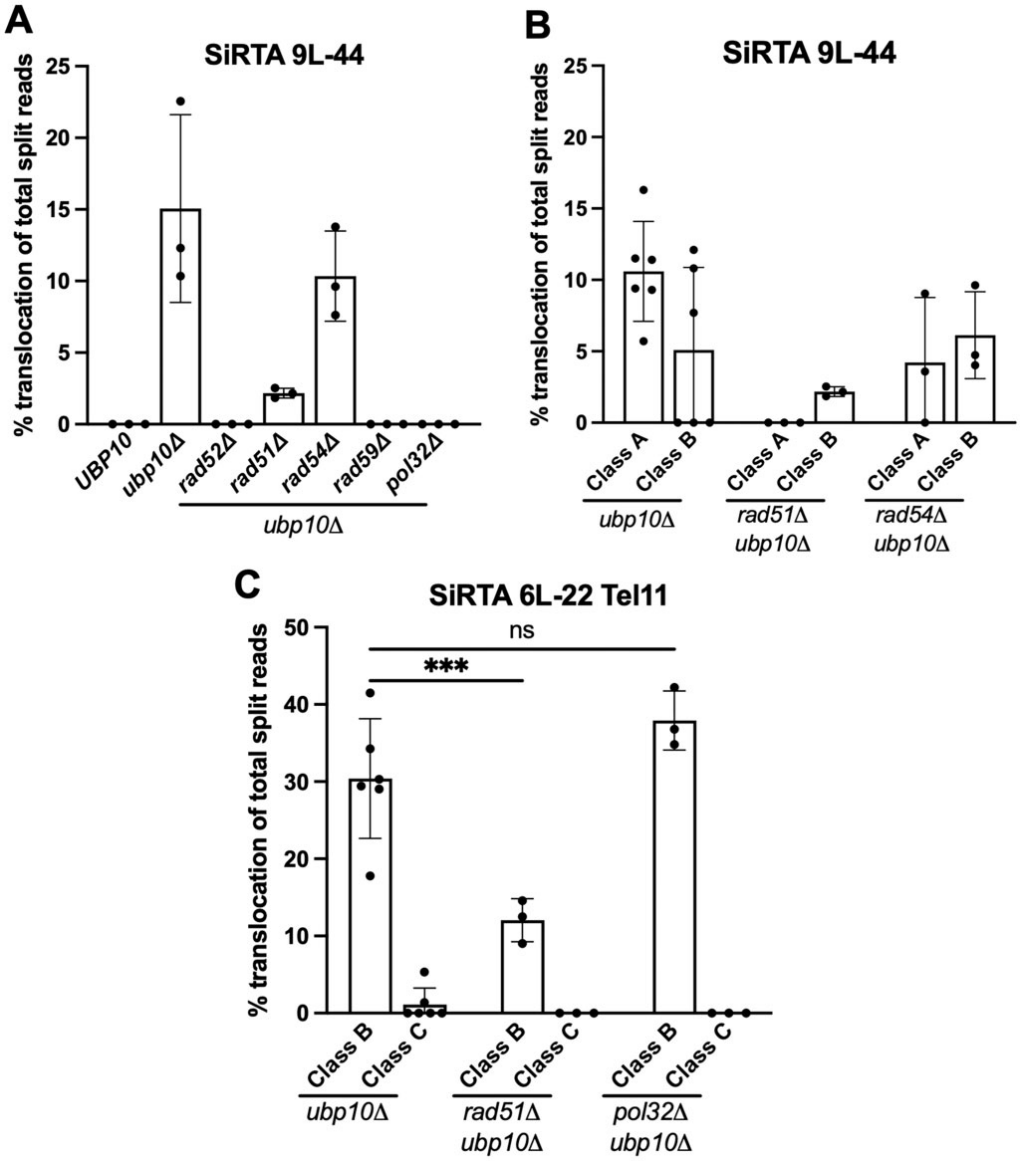
Class B translocations at SiRTAs occur independently of *RAD51* but differ in their dependencies on *POL32*. (**A**) The percentage of translocation split reads out of total split reads is shown at SiRTA 9L-44 for each strain indicated. Data are from three pools of 30 GCR events. (**B**) Of the strains that exhibited translocation events at SiRTA 9L-44, translocation split reads were assigned as Class A or Class B translocations. Class C translocations were not observed. Data for the *ubp10Δ* strain are repeated from Fig. 6B for comparison. (**C**) Translocation split reads were assigned as Class B or Class C at SiRTA 6L-22 Tel11 for each strain indicated. Data for SiRTA 6L-22 Tel11 *ubp10Δ* include the data from Fig. 6D and three additional replicates. ***P < .0001. Two-way ANOVA with Šídák’s multiple comparisons test.

We examined the requirements for translocations/deletions at SiRTA 9L-44 in the context of *UBP10* loss. As expected, deletion of *RAD52*, broadly required for HR, eliminates chromosome rearrangements at the SiRTA (Fig. 7A). In contrast, chromosome rearrangements at SiRTA 9L-44 are reduced, but not eliminated, by deletion of *RAD51* (Fig. 7A). Upon closer inspection, the remaining rearrangements in the *ubp10Δ rad51Δ* strain are exclusively Class B, suggesting that Class A events require Rad51, but Class B do not (Fig. 7B). We confirmed the latter result at SiRTA 6L-22 Tel11, where nearly all rearrangements in the absence of *UBP10* are Class B, accounting for ∼30% of total GCR events (Fig. 6D). While the frequency is reduced upon additional deletion of *RAD51* (to ~40% of that in the *RAD51 ubp10Δ* strain), Class B translocations clearly persist (Fig. 7C and Supplementary Fig. S10B), confirming that these events can occur through a *RAD51*-independent mechanism. Both Class A and Class B events at SiRTA 9L-44 require *RAD59* but are unaffected by deletion of *RAD54* (Fig. 7A and B). As expected, *dn*TA events persist at SiRTA 9L-44 in the absence of *RAD52* (Supplementary Fig. S10A), although *dn*TA events are reduced after deletion of *RAD51*, as we previously reported [26].

In the experiments described here, we select for clones that have lost sequences distal to the HOcs. Therefore, true translocations recovered in this assay are non-reciprocal and likely arise through BIR, a mechanism that operates when only one end of a DSB can productively engage in repair [13, 38, 39]. The requirement for *RAD52* is consistent with, but not diagnostic of, this type of repair. To evaluate the involvement of BIR, we deleted the nonessential subunit of Polδ (encoded by *POL32*), which is required for successful completion of BIR [40, 41]. At SiRTA 9L-44, Class A and Class B events are abolished in the *pol32Δ ubp10Δ* strain (Fig. 7A). We then examined the effect of deleting *POL32* and *UBP10* at SiRTA 6L-22 Tel11 and were surprised to find that the frequency of Class B events at SiRTA 6L-22 Tel11 is unaffected by deletion of *POL32* (Fig. 7C). It is unclear whether Class B events on chromosome VI are fundamentally different from those on chromosome IX or whether the high efficiency of events on chromosome VI masks a partial requirement for Pol32 (see “Discussion” section).

### Sir4 and Sir2 are required for a subset of subtelomeric translocations

The observation that most rearrangements in the *ubp10Δ* strain involve subtelomeric sequences suggests a potential mechanism for Ubp10 action. Yeast subtelomeric regions are transcriptionally silenced by recruitment of the SIR proteins [42, 43]. Ubp10 physically interacts with Sir4 to deubiquitinate H2B, promoting heterochromatin formation at telomeric loci [44, 45]. In the absence of *UBP10*, disruption of subtelomeric heterochromatin may provide a favorable substrate for recombination. If true, deletion of *SIR4* should promote rearrangements at SiRTAs, similar to deletion of *UBP10*. In contrast, we observe only *dn*TA events at SiRTA 9L-44 in the absence of *SIR4* (Supplementary Fig. S10C), indicating that loss of telomeric silencing alone does not explain the phenotype of the *ubp10Δ* strain.

**Figure 8.**
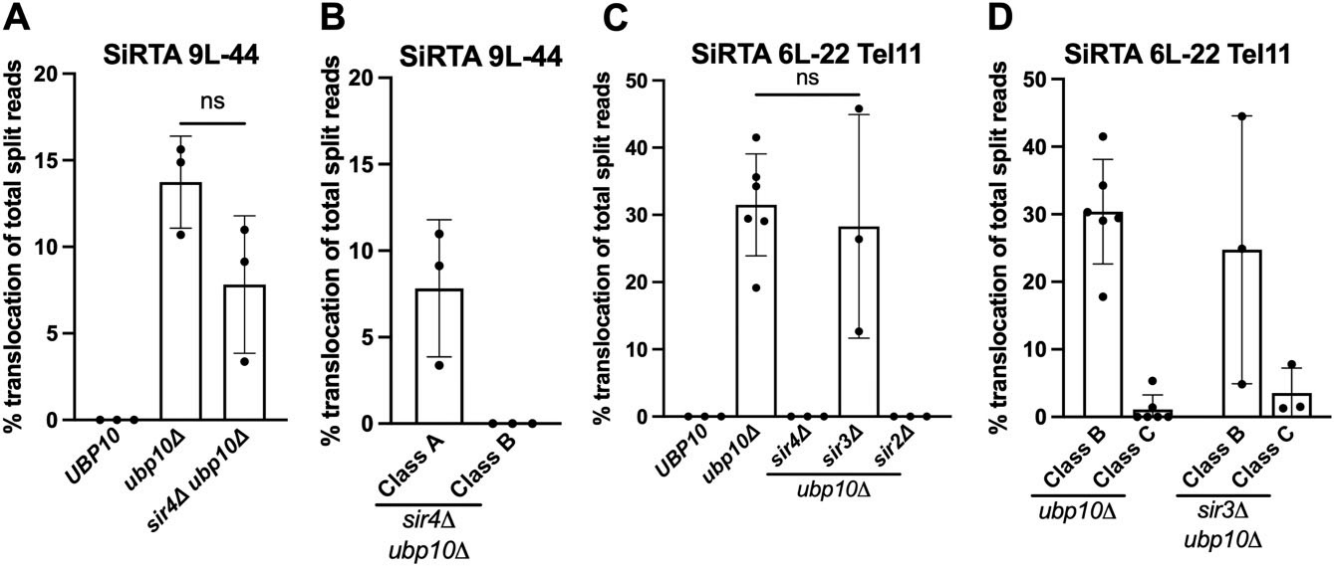
Class B translocations require Sir4 and Sir2. (**A**) Percentages of translocation split reads (of total split reads) are shown for the indicated strains. Data are from 3 pools of 30 GCR events. *P < .05 by ANOVA with Dunnett’s multiple comparisons test. (**B**) Translocation split reads observed at SiRTA 9L-44 in the *sir4Δ ubp10Δ* strain were divided into their respective class. (**C**) Percentages of translocation split reads (of total split reads) are shown at SiRTA 6L-22 Tel11 for the indicated strains. Data are from three pools of 30 GCR events. Data for the *ubp10Δ* strain are from Fig. 7C. ANOVA with Dunnett’s multiple comparisons test. (**D**) Translocation split reads observed at SiRTA 6L-22 Tel11 in the *sir3Δ ubp10Δ* strain were divided into their respective classes. Data for SiRTA 6L-22 Tel11 *ubp10Δ* are from 7C.

As a control, we deleted *SIR4* in the *ubp10Δ* background. Although the overall frequency of rearrangements at SiRTA 9L-44 was unchanged, Class B events were specifically eliminated (Fig. 8A and B). To examine the effect on Class B events more closely, we examined the effect of deleting the *SIR* genes on rearrangements at SiRTA 6L-22 Tel11. Deletion of *SIR2, SIR3*, or *SIR4*, all of which are required for telomeric silencing, has no effect on the frequency or type of rearrangements at SiRTA 6L-22 Tel11 in the *UBP10* background (Supplementary Fig. S10D). However, in the *upb10Δ* strain, Class B events at SiRTA 6L-22 Tel11 are eliminated by deletion of *SIR4* or *SIR2* (Fig. 8C), although *dn*TA events increase (Supplementary Fig. S10D). Surprisingly, Class B rearrangements persist in the absence of *SIR3* (Fig. 8C and D; Supplementary Fig. S10D), indicating that the role of the SIR complex in telomeric silencing cannot explain the effect on these events. Loss of silencing at the mating type loci can indirectly affect DNA repair pathways by activating gene expression patterns typical of diploid cells [46]. However, in addition to the lack of effect with *SIR3* deletion, the strains that we use for these experiments contain deletions at the silent mating loci [24, 26] that further rule out this explanation for the loss of Class B translocations upon deletion of *SIR2* or *SIR4*.

## Discussion

We and others have reported that telomere-like sequences in the yeast genome stimulate chromosome truncation through *dn*TA, a phenomenon also observed in some human diseases characterized by recurrent telomere addition events [20, 21, 24, 25]. Sites functioning as hotspots for telomere addition (SiRTAs) contain TG-rich sequences to which Cdc13 binds following resection of a DSB [24–26]. In this work, we characterize an unanticipated role of the ubiquitin protease, Ubp10, in modulating the type of repair that occurs at SiRTAs. Loss of *UBP10* has little or no impact on the probability of GCR formation following a DSB (total GCR frequency) (Supplementary Table S6), and the “efficiency” of the SiRTA (measured as the fraction of total GCR events that occur at the SiRTA) remains largely unchanged (Figs 2A, C and 5A). However, *dn*TA at the SiRTA decreases, while other types of rearrangements, most predominantly translocations to subtelomeric regions, rise in abundance. We infer that, in wild-type cells, Ubp10 represses successful engagement of SiRTAs with repair pathways that lead to chromosome rearrangements. While Ubp10 may promote *dn*TA at SiRTAs, preventing rearrangements through the deletion of *RAD52* restores *dn*TA in the *ubp10Δ* background (Supplementary Fig. S10A), suggesting that any apparent effect on telomere addition reflects competition between alternative repair pathways.

### How does Cdc13 promote subtelomeric rearrangements at SiRTAs?

Multiple lines of evidence suggest that association of Cdc13 with the SiRTA is required to stimulate subtelomeric rearrangements in the absence of *UBP10*. The chromosomal locations at which rearrangements are observed in the absence of *UBP10* correlate with sites at which telomere addition normally occurs, most prominently at SiRTAs (Fig. 3). The exception occurs adjacent to the HOcs, where telomere addition is likely stimulated by the Ku heterodimer [24], rather than Cdc13 [36]. Mutation of the SiRTA Stim sequence, previously shown to bind Cdc13, strongly reduces both *dn*TA and other rearrangements at two different SiRTAs (Fig. 4). Additionally, integration of a high-affinity Cdc13 binding site at an otherwise extremely weak SiRTA strongly stimulates both *dn*TA and translocations in the absence of *UBP10* (Fig. 5). These results imply a previously unanticipated role of Cdc13 in facilitating rearrangement events involving SiRTAs.

The requirement for Cdc13 to stimulate rearrangements could be explained if abortive telomere addition at the SiRTA, catalyzed by Cdc13-mediated recruitment of telomerase, occurs prior to engagement in a secondary repair event. In the case of the Class C rearrangements that contain telomeric repeats at the breakpoint, this is a likely explanation (Supplementary Fig. S9C). In contrast, Class A and B translocations lack TG_1-3_ sequences, although it is formally possible that telomeric sequences are removed prior to resolution of the repair event. For this reason, we favor the idea that association of Cdc13 with SiRTAs promotes rearrangements in the *ubp10Δ* strain by facilitating localization of the broken end to the nuclear periphery. Cdc13 is implicated in the sequestration of persistent DSBs through association with the telomerase component Est1, which in turn supports the occurrence of spontaneous GCRs [47, 48]. Since telomeres also cluster at the nuclear periphery, colocalization of breaks and telomeric/subtelomeric sequences may promote interactions conducive for the formation of rearrangements [47, 49–51]. A fraction of rearrangement breakpoints map proximal to the SiRTA 5L-35 Stim sequence and to the inserted Cdc13 binding site at SiRTA 6L-22 Tel11, despite being stimulated by those sequences. Since *dn*TA events always occur distal to the Stim sequence, this pattern is inconsistent with the idea that telomere addition *per se* is required and supports a model where the break is relocalized prior to additional processing that removes the sequence originally bound by Cdc13.

The dependency of Class B rearrangements on Sir4 and Sir2 is consistent with this model. Sir4 plays a key role in anchoring subtelomeric chromatin to the nuclear periphery [47, 51, 52], suggesting that spatial proximity between the SiRTA and the subtelomeric ITS within the same nuclear subcompartment enhances the likelihood of a rearrangement [47, 53]. The differential effects of Sir2 and Sir3 are less easily explained, although Sir3 has been reported to be dispensable for the localization of telomeres to the nuclear periphery [50]. It is possible that Sir2 (in complex with Sir4) stabilizes the association of the broken end with the nuclear periphery and with subtelomeric chromatin, creating an environment conducive for recombination with telomeric sequences. Microhomology involved in Class A rearrangements may lessen the dependency on this effect.

### Variable genetic requirements suggest that multiple repair pathways may act at SiRTAs

The rearrangements observed at SiRTA 9L-44 in the absence of *UBP10* are eliminated by deletion of *RAD52* (Fig. 7A), suggesting repair through HR. The presence of either limited microhomology (Class A) or no microhomology (Class B) at the breakpoint predicts independence from Rad51 [16, 54]. Indeed, Class B rearrangements persist in the absence of Rad54 or Rad51 (although at reduced frequency) and require Rad59, consistent with repair by either SSA or Rad51-independent BIR (see below) [13, 54–56]. Class A rearrangements likewise require Rad59 and are independent of Rad54 but, surprisingly, require Rad51 (Fig. 7). We speculate that the requirement for Rad51 arises from its role in promoting Cdc13 association with SiRTAs, rather than a direct role in recombination. We previously reported that deletion of *RAD51* impairs *dn*TA at SiRTAs ([26]; see also, Supplementary Figs 2D and 10B) and is associated with reduced recruitment of Cdc13 following a DSB, likely due to competition between Cdc13 and RPA at the SiRTA [26]. Since a Cdc13 binding site is necessary and sufficient to stimulate rearrangements at SiRTAs (Figs 4 and 5), decreased association of Cdc13 following loss of *RAD51* should reduce the probability of rearrangements in the *ubp10Δ* background. The differential impact of Rad51 deletion suggests that Class B rearrangements are more tolerant of reduced Cdc13 binding than Class A events.

SiRTA 9L-44 rearrangements are abolished by deletion of Pol32, consistent with the generation of these events by BIR, a pathway that facilitates the repair of one-ended DSBs by invading and copying (through conservative replication) a homologous sequence [13, 38, 39, 56]. For the Class B events, this result is expected. Subtelomeric sequences acquired by the Class B events must be acquired from a different chromosome end because the 5′-ATATATAT-3′ motif at the breakpoint is absent from the left telomere of chromosome IX. In contrast, Class A events most likely involve repair events between SiRTA 9L-44 and the chromosome IX subtelomere, although we cannot rule out involvement of identical sequences on chromosome X. The requirement for Pol32 argues that Class A events occur through BIR rather than SSA, with duplication of distal sequences occurring in *cis* prior to degradation of the chromosome terminus [13], [40, 57]. A similar preference for BIR between repeated sequences has been observed in mammalian cells when the break is located asymmetrically between the repeats [13, 39].

In contrast to the observations at SiRTA 9L-44, Class B events at SiRTA 6L-22 Tel11 persist in the *pol32Δ ubp10Δ* background (Fig. 7C). BIR initiates and synthesis can continue as much as 15 kb in the absence of Pol32 [41]. Given the subtelomeric locations of the target sequence for Class B events, replication extends 4.5–7 kb to reach the chromosome end and therefore may not rely strongly on Pol32. However, it is unclear why this effect would differ at 9L-44 and 6L-22 Tel11. The chromosome VI left subtelomere contains an ITS followed by the 5′-ATATATAT-3′ motif found at the breakpoint of Class B events. Therefore, rearrangements on chromosome VI may be large deletions and could occur through SSA, accounting for independence from Pol32 function [15]. Resolution of these possibilities will require additional genetic analysis, combined with long-read sequencing to distinguish deletions from true translocations.

### Translocations at SiRTAs may mimic events in alternative lengthening of telomeres

Rearrangements observed at SiRTAs in the absence of *UBP10* are reminiscent of those produced by ALT, a pathway utilized by 10%–15% of cancer cells to maintain telomere function [17, 18]. Recombination as a mechanism to stabilize chromosome ends in the absence of telomerase was first characterized in *S. cerevisiae*, and budding yeast serves as a robust model to study ALT [17, 18]. However, analysis of initial ALT survivor formation remains challenging due to the genomic instability generated in cells lacking telomerase. Translocations formed at SiRTAs may provide a model to study the initial step of ALT-survivor formation. Type I ALT survivors acquire multiple Y′ elements from various subtelomeric donors, a process that may be analogous to acquisition of telomeric and Y′ element sequences in Class C events observed at SiRTAs 14L-35 and 6L-22 Tel11 (Figs 2E and 6D; Supplementary Fig. 9C) [18]. Class B events may represent a previously unrecognized mechanism through which Y′ elements can be acquired in the absence of obvious microhomology. Further characterization and quantification of both types of events will require long-read sequencing to clarify where recombination initiates (i.e. ITS versus terminal repeat in the case of Class C events) and whether template switching (TS) between Y′ elements is involved in the successful resolution of these events [58].

### Mechanism of Ubp10 suppression of subtelomeric rearrangements

SiRTAs have the capacity to facilitate subtelomeric rearrangements, but that propensity is normally suppressed by the deubiquitylase activity of Ubp10 (Fig. 2B). Ubp10 counteracts ubiquitylation of histone 2B (H2B) and proliferating cell nuclear antigen (PCNA), both of which are associated with DNA damage signaling pathways [59–64]. We find that loss of telomeric silencing (a consequence of H2B ubiquitylation) does not explain the phenotype of the *ubp10Δ* strain (Fig. 8 and Supplementary Fig. S10C and D), although we cannot rule out the possibility that rearrangements are facilitated in other ways by increased levels of ubiquitinated H2B^K123^.

Ubiquitin modification of PCNA at lysine 164 serves as a nexus of multiple DNA damage tolerance pathways [19, 65]. Monoubiquitination promotes translesion synthesis through recruitment of damage-tolerant polymerases capable of bypassing modified bases at the replication fork [61, 65]. Extension to a K63-linked polyubiquitin chain stimulates error-free TS, a mechanism that genetically resembles BIR [19, 61, 65]. The failure to appropriately regulate ubPCNA levels in the absence of Ubp10 may facilitate the formation of rearrangements through upregulation of TS activity, promoting the initiation and/or successful completion of repair. Of note, Ubp10 promotes unloading of PCNA during lagging strand replication and is required for normal Okazaki fragment processing [64, 66]. Since Rad51-independent BIR and SSA both require ssDNA to circumvent the need for strand invasion by Rad51 [16, 39, 58], increased generation of ssDNA within subtelomeres in the *ubp10Δ* strain could facilitate repair at SiRTAs, a mechanism that warrants further investigation. Together, our study describes a role of Ubp10 as a regulator of DNA repair type at interstitial telomere-like sites, highlighting the interplay between telomere maintenance mechanisms and genomic stability.

## Supplementary Material

gkaf1373_Supplemental_Files

## Data Availability

Strains and plasmids are available upon request. The data presented in this article are available in the article, in its online Supplementary material, or will be shared upon reasonable request to the corresponding author. Sequencing data are available from the NIH Sequence Read Archive (SRA) under BioProject ID: PRJNA1279155.

## Notes

Current address: School of Nursing & Health Sciences, Merrimack College, North Andover, MA 01845, United States
